# In Silico Identification of LSD1 Inhibition-Responsive Targets in Small Cell Lung Cancer

**DOI:** 10.3390/bioengineering12050504

**Published:** 2025-05-10

**Authors:** Ihsan Nalkiran, Hatice Sevim Nalkiran, Neslihan Ozcelik, Mehmet Kivrak

**Affiliations:** 1 Department of Medical Biology, Faculty of Medicine, Recep Tayyip Erdogan University, 53020 Rize, Türkiye; ihsan.nalkiran@erdogan.edu.tr (I.N.); hatice.sevim@erdogan.edu.tr (H.S.N.); 2 Department of Chest Diseases, Faculty of Medicine, Recep Tayyip Erdogan University, 53020 Rize, Türkiye; neslihan.ozcelik@erdogan.edu.tr; 3 Department of Biostatistics and Medical Informatics, Faculty of Medicine, Recep Tayyip Erdogan University, 53020 Rize, Türkiye

**Keywords:** small cell lung cancer, LSD1 inhibitor, differentially expressed genes, bioinformatics, RG6016, ORY-1001, molecular docking

## Abstract

Small cell lung cancer (SCLC) is an aggressive neuroendocrine malignancy characterized by rapid progression, high metastatic potential, and limited therapeutic options. Lysine-specific demethylase 1 (LSD1) has been identified as a promising epigenetic target in SCLC. RG6016 (ORY-1001) is a selective LSD1 inhibitor currently under clinical investigation for its antitumor activity. In this study, publicly available RNA-Seq datasets from SCLC patient-derived xenograft (PDX) models treated with RG6016 were reanalyzed using bioinformatic approaches. Differential gene expression analysis was conducted to identify genes responsive to LSD1 inhibition. Candidate genes showing significant downregulation were further evaluated by molecular docking to assess their potential interaction with RG6016. The analysis identified a set of differentially expressed genes following RG6016 treatment, including notable downregulation of MYC, UCHL1, and TSPAN8. In silico molecular docking revealed favorable docking poses between RG6016 and the proteins encoded by these genes, suggesting potential direct or indirect targeting. These findings support a broader mechanism of action for RG6016 beyond its known interaction with LSD1. This study demonstrates that RG6016 may exert its antitumor effects through the modulation of additional molecular targets such as MYC, UCHL1, and TSPAN8 in SCLC. The combined bioinformatic and molecular docking analyses provide new insights into the potential multi-target profile of RG6016 and indicate the need for further experimental validation.

## 1. Introduction

Small cell lung carcinoma (SCLC) accounts for approximately 15% of all lung cancers, which are among the leading causes of cancer-related deaths worldwide [1]. SCLC is strongly linked to tobacco use [2] and is characterized by a high proliferation rate, early metastatic spread, and extremely poor prognosis [1]. The aggressive clinical course of SCLC is largely attributed to its high mutational burden and genomic instability [3].

The biological heterogeneity of SCLC, despite its relatively uniform histological appearance, has emerged as a significant subject of extensive research in recent years [4]. In addition to intertumoral heterogeneity in molecular alterations and the expression of key transcription factors such as TP53, RB1, and MYC, SCLC also exhibits significant intratumoral heterogeneity. This is reflected in the coexistence of cancer cell populations with varying drug sensitivities and resistance mechanisms, which may contribute to the development of chemoresistance, disease progression, and metastasis [5,6]. SCLC is considered one of the most highly metastatic solid tumors, with widespread metastases to the lymph nodes, brain, liver, and bones commonly present at the time of diagnosis in the majority of patients [7]. The high metastatic potential of SCLC is further supported by the high number of circulating tumor cells (CTCs) observed in these patients, offering a unique opportunity for metastatic dissemination [8].

Lysine-specific demethylase 1 (LSD1) functions as a histone demethylase that removes H3K4 mono- and di-methylation transcription marks [9]. Small-molecule inhibitors targeting LSD1 have demonstrated promising antitumor activity in preclinical models of malignancies such as acute myeloid leukemia (AML), SCLC, and medulloblastoma [10,11,12,13]. In addition to inhibiting its enzymatic activity, some LSD1 inhibitors also function by disrupting the interaction between LSD1 and SNAG domain-containing proteins [14], as well as its interaction with chromatin [10]. To date, irreversible LSD1 inhibitors such as tranylcypromine, IMG-7289, INCB059872, GSK-2879552, ORY-1001 (RG6016), ORY-2001, TAK-418, and LH-1802, as well as reversible inhibitors such as SP-2577 and CC-9001, have entered clinical trials [15]. RG6016 is a highly potent and selective covalent LSD1 inhibitor with high selectivity against related flavin adenine dinucleotide (FAD) that has been evaluated in phase I/II clinical trials. This compound has been tested in patients with relapsed or refractory SCLC and AML for its safety profile, pharmacodynamic effects, and maximum tolerated dose [16,17,18].

This study aims to investigate the selective efficacy of the LSD1 inhibitor RG6016 across SCLC subgroups and to elucidate the molecular mechanisms supporting this efficacy. To this end, RNA-seq data from seven ex vivo cultured SCLC patient-derived xenograft (PDX) samples and control groups, obtained from the NCBI-GEO (Gene Expression Omnibus) database under the study titled ‘Targeting NOTCH activation in small cell lung cancer through LSD1 inhibition’, were analyzed using bioinformatics methods. Through the analysis of transcriptomic data obtained from the GEO database, the expression profiles of target genes responsive to LSD1 inhibition were identified, and molecular docking analyses were performed to evaluate potential target genes. Favorable docking poses between RG6016 and the proteins encoded by these target genes were evaluated through molecular docking studies. This study aims to contribute to a better understanding of the therapeutic potential and mechanisms of LSD1 inhibition in SCLC.

## 2. Material and Methods

### 2.1. Bioinformatic Processing of Public RNA-Seq Data

**Figure 1:**
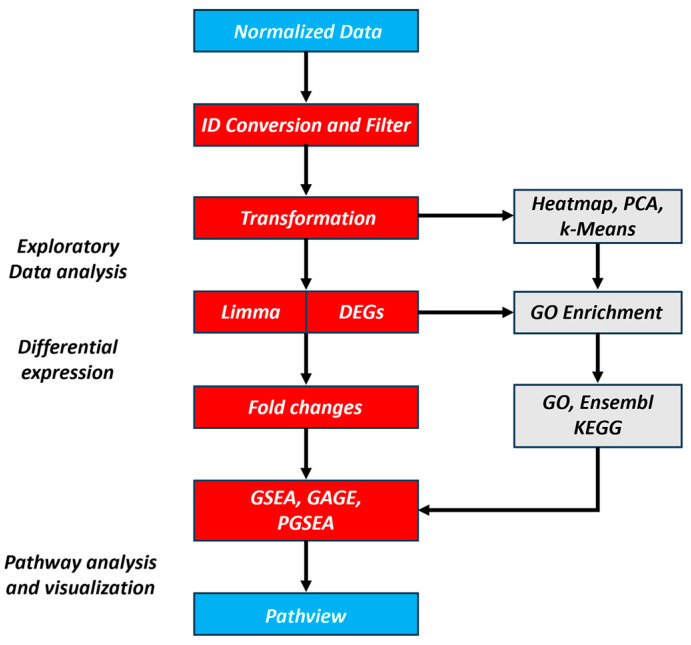
Workflow of normalized expression and RNA-seq read count analysis.

This study reanalyzed RNA-Seq datasets from the publication ‘Targeting NOTCH activation in small cell lung cancer through LSD1 inhibition’ [19] available in the NCBI GEO database, using bioinformatics methods to identify target genes associated with treatment response as potential biomarkers. The referenced study investigated the selective efficacy of the LSD1 inhibitor RG6016 across SCLC PDX models. In this study, we reanalyzed publicly available RNA-Seq data generated from PDX models of SCLC, which were established from CTCs or tumor tissues of patients with extensive-stage disease and treated with the LSD1 inhibitor RG6016; the models were propagated in NSG mice and subjected to drug treatment protocols involving oral administration of RG6016 or saline control, enabling the investigation of transcriptional responses to LSD1 inhibition. The RNA-Seq data have been made publicly available in the Gene Expression Omnibus database under the accession numbers GSE103095, GSE103096, and GSE103097 [19]. In the present study, the downloaded RNA-Seq data were re-evaluated using bioinformatics approaches, and analyses were conducted to identify potential target genes. RNA-Seq data from a total of 14 samples—including 7 ex vivo cultured SCLC samples treated with RG6016 and corresponding control groups—were examined in detail using bioinformatics methods (Table 1). During the data analysis process, dimensionality reduction was performed on the raw dataset containing 35,230 genes by applying Principal Component Analysis (PCA), reducing the number of genes to 18,539. Subsequently, the processed data matrix containing normalized gene expression levels was analyzed using R (version 3.20) and Bioconductor (version 4.4.0) packages for exploratory analysis, differential gene expression (DEG) identification, and pathway enrichment analyses. The selective effects of the LSD1 inhibitor on gene expression were identified, and the underlying molecular mechanisms were analyzed. The bioinformatics analysis of RNA-Seq data enabled a detailed characterization of the genetic and biological profiles of each subgroup, resulting in the identification of candidate target genes [20]. Figure 1 provides a flowchart summarizing the analysis process.

### 2.2. Data Preprocessing

**Figure 2:**
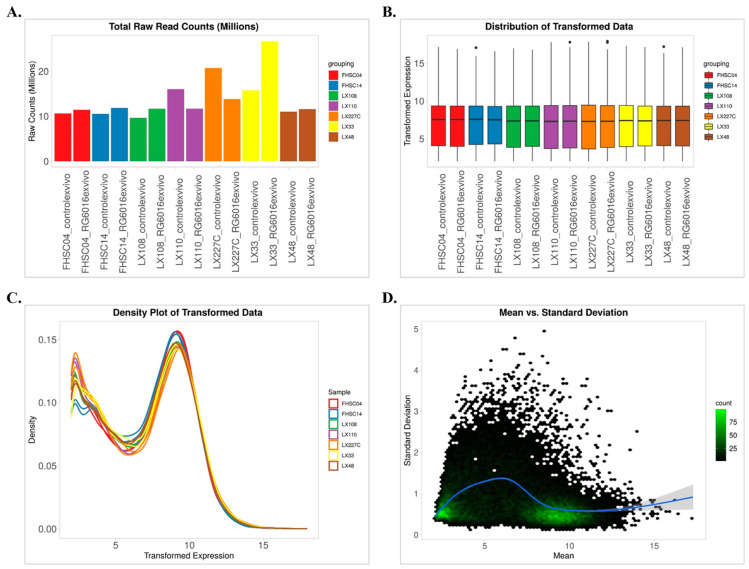
An overview of RNA sequencing data in the control and RG6016-treated PDX samples. (**A**) The total raw read counts (in millions) across all PDX samples are visualized. Sequencing depth was consistently maintained across treatment conditions. (**B**) The boxplot of log_2_-transformed gene expression data shows similar distributions across samples, confirming successful normalization. (**C**) The density plot of the transformed data indicates consistent gene expression distributions across all samples. (**D**) The dispersion plot visualizes the relationship between the mean expression levels and standard deviations of genes, highlighting the variance in gene expression. The blue line represents the trend between mean and standard deviation, while the grey shadow indicates the confidence interval around the trend. These analyses support the reliability of the data and the success of the normalization process.

During the data preprocessing stage, gene identifiers were matched with a reference database to enable automatic conversion and species annotation. Genes with minimal downregulation were excluded from the analysis based on statistical thresholds determined using a threshold-based Wald test (FDR ≤ 0.1 and minimum fold change ≥ 2). This selection was made to enhance the reliability of the differential expression analysis by considering the experimental design, samples, and treatment conditions. The data were appropriately transformed using various methods. Analysis using the edgeR library in R (version 3.20) and Bioconductor (version 4.4.0) revealed that the data distribution for both control and treatment groups across all samples was skewed. This skewness was corrected by applying a log_2_ transformation, which approximated a normal distribution. The transformation preserved small values while reducing variability across all samples and had a notable impact on downregulated genes. Raw read counts in the cells reflected the quantity of sequencing reads generated under different experimental conditions. However, variations observed between samples were indicative of possible technical variations arising from sample preparation and sequencing processes. These differences were assessed to determine the presence of a potential batch effect, and normalization procedures were applied accordingly (Figure 2A). Following normalization and transformation, the distribution of gene expression levels was examined, and it was observed that systematic differences between samples were largely corrected. This process ensured the reduction in inter-sample variance and enabled the comparability of gene expression levels (Figure 2B). The distribution of transformed gene expression data between control and treatment groups was examined using density plots, allowing for the visualization of expression patterns across the dataset (Figure 2C). Density plots were chosen as they provide a continuous curve representation of the overall distribution of gene expression levels, helping detect variation and potential systematic changes between groups. Additionally, boxplot analyses were performed to further enhance the comparability of the dataset. The variance of gene expression levels was assessed, and genes were filtered based on their statistical reliability and suitability for differential expression analysis. FDR ≤ 0.1 and minimum fold change ≥ 2 were used as threshold values for gene filtering (Figure 2D). As a result, only genes that showed statistically significant and biologically relevant changes were included in the analysis.

### 2.3. Principal Component Analysis

**Figure 3:**
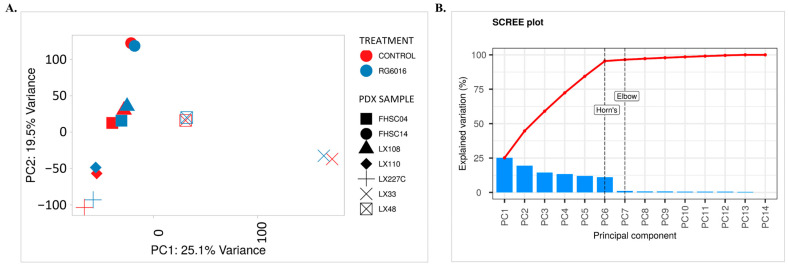
Investigation of the effects of RG6016 on PDX samples using PCA. (**A**) PCA was performed on the gene expression matrix obtained from RNA-seq data. The PCA score plot shows how the control and RG6016 treatment groups in different PDX samples are distributed along the principal components (PC1 and PC2). Treatment groups are represented by colors (red: control; blue: the RG6016), while PDX samples are indicated by different shapes. (**B**) The scree plot illustrates the contribution of each principal component to the explained variance. PC1 and PC2 account for 25.1% and 19.5% of the total variance, respectively, making them the components that carry the most information. The elbow point helps identify the most meaningful components, and the optimal number of components was further supported by Horn’s parallel analysis method. The red line represents the cumulative proportion of explained variance across the principal components.

In this study, Principal Component Analysis (PCA) was applied to reduce the dimensionality of the dataset and to identify underlying patterns [21]. PCA was performed on the gene expression matrix derived from RNA sequencing data to examine the effects of control and RG6016 treatments on different SCLC PDX models. The PCA score plot presented in Figure 3A reveals a separation of samples based on treatment status and PDX model. However, some samples from the control and RG6016 treatment groups did not fully conform to the expected clustering pattern. This discrepancy is associated with the distinct biological responses of the PDX models to RG6016 treatment. Since RNA-Seq data reflect the biological heterogeneity of the cells, considerable variation in gene expression profiles was observed, leading to a wide distribution in the PCA plot. During the analysis process, it was determined that subdividing the samples and optimizing the normalization steps prior to PCA were necessary to achieve optimal results. Following this optimization, the distribution of the subgroups was arranged as shown in Figure 3A. The PCA score plot in Figure 3A confirms the separation of samples based on treatment groups and PDX samples. The first principal component (PC1), which accounts for 25.1% of the variance, primarily represents differences between PDX samples, while PC2 and PC3, accounting for 19.5% and 14.4% of the variance, respectively, contribute to the separation within each treatment group. As shown in Figure 3B, the first three principal components explain 59% of the total variation in the dataset, while the contribution of the remaining components is minimal. These findings demonstrate that PCA effectively distinguishes gene expression profiles between RG6016-treated and control groups, and that the variance captured by PC1, PC2, and PC3 reflects differences in cellular responses to the treatment.

### 2.4. Molecular Docking

**Figure 4:**
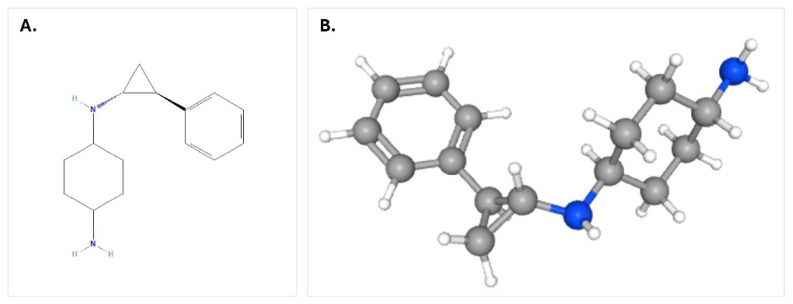
Structure of RG6016 molecule: (**A**) 2D chemical structure, (**B**) 3D molecular model.

In this study, potential target proteins identified through bioinformatic analyses for SCLC subtypes were evaluated for their possible binding interactions with the LSD1 inhibitor RG6016 using molecular docking methods. By examining the favorable docking poses of RG6016 with different proteins, the aim was to gain structural insights into the molecular mechanisms underlying the therapeutic effects of LSD1 inhibition in SCLC. The chemical structure of RG6016, used as the ligand, was obtained in PDB format from the PubChem database (CID: 71543365) (Figure 4). The molecule underwent energy minimization using UCSF Chimera 1.18 software [22] and was prepared for molecular docking using AutoDock Tools software (Version: 1.5.7) [23].

The three-dimensional structures of the proteins, based on the products of the target genes, were primarily obtained from the Protein Data Bank (PDB) database [24]. For proteins without available crystal structures, models were retrieved from the AlphaFold Protein Structure Database (https://alphafold.ebi.ac.uk/) (accessed on 23 March 2025) [25]. In the docking analyses, the crystal structure of the LSD1/FAD complex (PDB ID: 2V1D), the known target of RG6016, was used as a positive control. The docking of RG6016 to LSD1 was performed as a positive control to validate the docking workflow, given that the covalent interaction between RG6016 and LSD1 is experimentally established in prior studies. The binding score for LSD1 was not used as a quantitative reference, but rather to confirm the plausibility of the docking protocol against a known target. MYC (PDB ID: 1NKP) and UCHL1 (PDB ID: 2ETL) crystal structures were also included in the analysis. The remaining protein models were obtained from the AlphaFold Protein Structure Database and included the following: TSPAN8 (AF-P19075-F1-model_v4), BEX1 (AF-Q9HBH7-F1-model_v4), BEX3 (AF-Q00994-F1-model_v4), CALCA (AF-P01258-F1-model_v4), CD99 (AF-P14209-F1-model_v4), IRX2 (AF-Q9BZI1-F1-model_v4), MAGED4 (AF-Q96JG8-F1-model_v4), OLFM1 (AF-Q99784-F1-model_v4), SEZ6L (AF-Q9BYH1-F1-model_v4), TFF3 (AF-Q07654-F1-model_v4), and SPOCK1 (AF-Q08629-F1-model_v4). The structural quality of each model was assessed based on the average predicted Local Distance Difference Test (pLDDT) scores provided by AlphaFold. These pLDDT scores serve as an internal confidence metric for specific regions of the model. Generally, pLDDT values above 70 indicate reliable backbone predictions, whereas lower scores may correspond to intrinsically disordered regions or flexible loops. In this study, only human-derived protein models were selected for docking analyses. As a result, although some proteins exhibited average pLDDT scores below 70, they were included in the analyses due to the lack of alternative suitable structures in the AlphaFold database. Notably, proteins such as TSPAN8 (88.12), OLFM1 (79.39), and TFF3 (80.13) exceeded the high-confidence threshold. In contrast, proteins including BEX1, BEX3, CALCA, CD99, IRX2, MAGED4, SEZ6L, and SPOCK1 were also considered despite lower pLDDT scores, as they were of human origin and relevant to the experimental context of this study. A summary of the corresponding UniProt identifiers and average pLDDT scores is provided in Table S1 [26].

All protein structures were prepared for docking analysis using AutoDock Tools (Version: 1.5.7) [23]. During this process, polar hydrogen atoms were added, Gasteiger charges and Kollman united atom charges were assigned, and water molecules present in crystal structures were removed. For each target protein, binding regions were defined using AutoDock Tools 1.5.7. Grid boxes were sized to encompass the potential binding sites, and center coordinates (X, Y, Z) were determined. Binding regions were identified by positioning the ligand within the presumed cavity, and the grid box center was determined based on the geometric location of the ligand as automatically suggested by AutoDock Tools [27]. Docking analyses were conducted for a total of 14 proteins, including the positive control LSD1/FAD complex and 13 target proteins identified through bioinformatics analyses. Specific grid coordinates and box sizes for each protein are detailed in Table 2. Docking studies were performed using AutoDock Vina (Version: 1.1.2) [28]. The binding energies (kcal/mol) of RG6016 to each target protein were calculated, and conformations with the lowest binding energies were selected for further analysis. The docking scores reported in this study reflect pose-fitting estimations based on the AutoDock Vina scoring function. These scores do not represent thermodynamic binding affinities and should not be interpreted as direct measures of binding energy. Ligand–protein interactions based on these conformations were analyzed using BIOVIA Discovery Studio 2024 Client (Dassault Systèmes, 2024).

## 3. Results

### 3.1. Correlation Matrix Among PDX Samples

**Figure 5:**
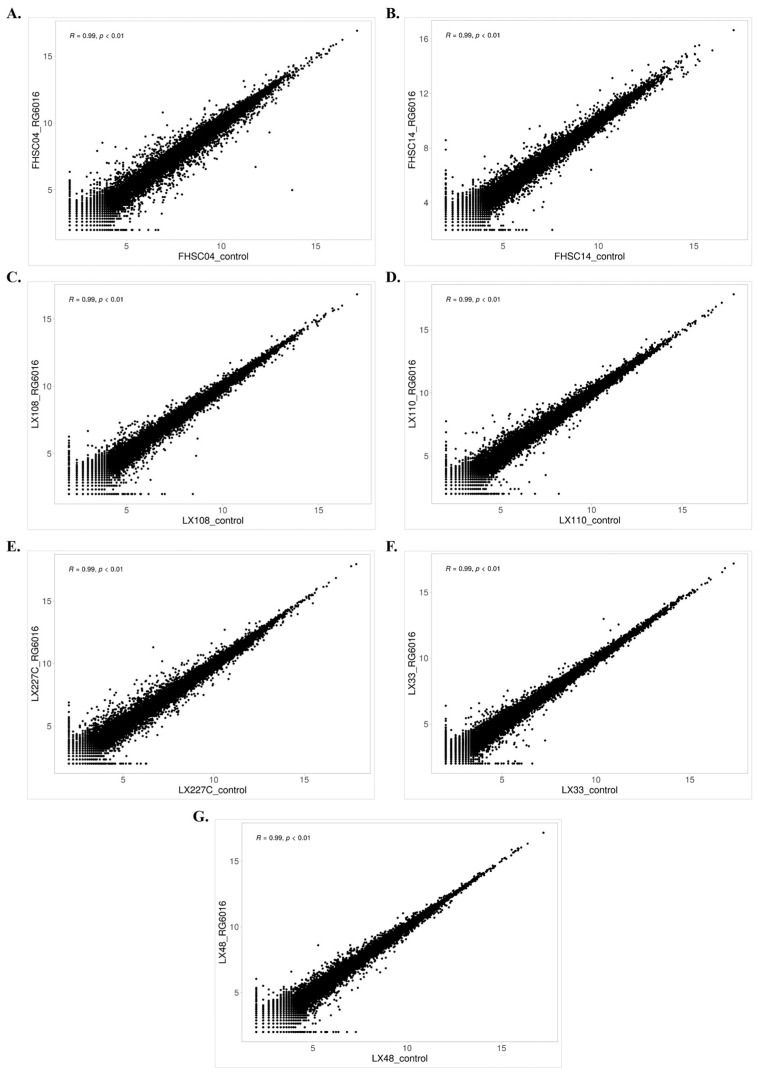
Scatter plots of log_2_-transformed gene expression data. Gene expression levels in control and RG6016-treated groups were compared for the following PDX samples: (**A**) FHSC04, (**B**) FHSC14, (**C**) LX108, (**D**) LX110, (**E**) LX227C, (**F**) LX33, and (**G**) LX48. Each point represents a gene, with the *X*-axis indicating the control group and the *Y*-axis indicating the treatment group.

The gene expression profiles of the control and RG6016-treated PDX samples were analyzed using log_2_-transformed values and scatter plots to evaluate linear relationships (Figure 5). For each biological replicate, Pearson correlation coefficients (r) and *p*-values were calculated to assess the strength of these relationships. Strong and positive correlations were observed between FHSC04-control and FHSC04-RG6016 (r = 0.99, *p* < 0.001), FHSC14-control and FHSC14-RG6016 (r = 0.99, *p* < 0.001), LX108-control and LX108-RG6016 (r = 0.99, *p* < 0.001), LX110-control and LX110-RG6016 (r = 0.99, *p* < 0.001), LX227C-control and LX227C-RG6016 (r = 0.99, *p* < 0.001), LX33-control and LX33-RG6016 (r = 0.99, *p* < 0.001), and LX48-control and LX48-RG6016 (r = 0.99, *p* < 0.001). The observed high positive correlations reflect a strong similarity and reproducibility of gene expression profiles across diverse PDX samples in both control and treatment conditions, emphasizing the reliability and consistency of the experimental data.

### 3.2. Differentially Expressed Genes2 (DEG2) Analysis

**Figure 6:**
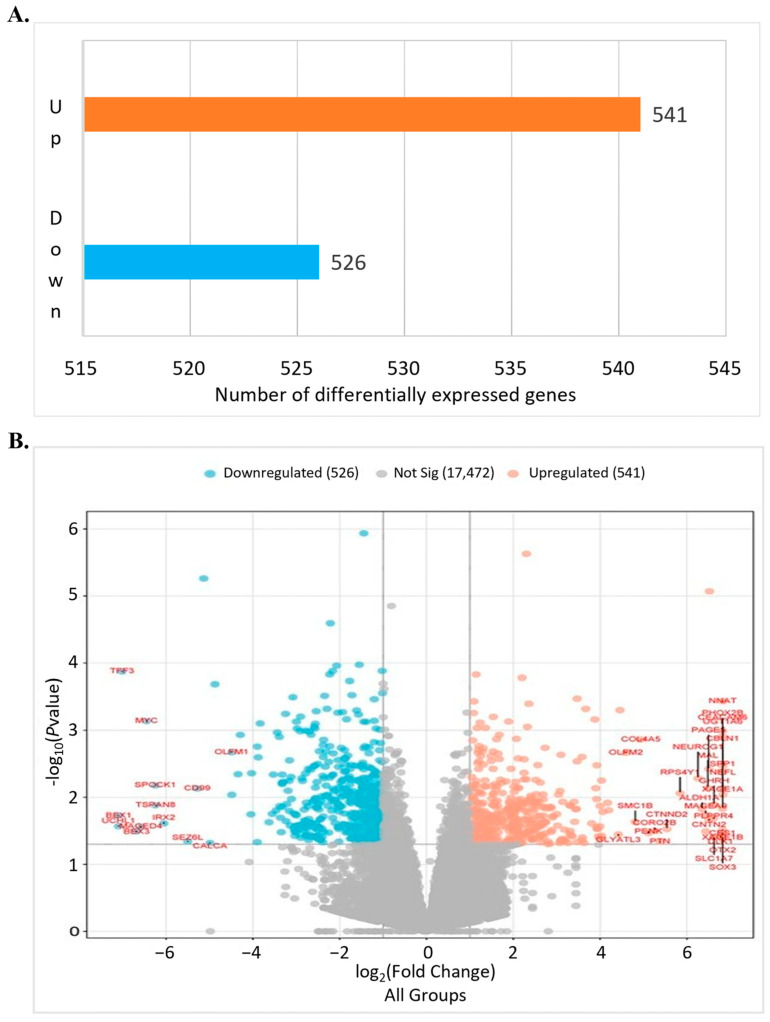
The number of DEGs and log_2_ fold change plots. The number of DEGs and the distribution of log_2_ fold changes identified in the differential expression analysis are visualized. In the experimental design, the gene expression profile of each PDX sample was compared against a general category comprising all PDX samples. (**A**) shows the number of genes differentially expressed between the control and RG6016 treatment groups. (**B**) The volcano plot illustrates the relationship between log_2_ fold change and adjusted *p*-value. The blue dots represent downregulated genes, the orange dots represent upregulated genes, and the gray dots indicate genes with no statistically significant change.

In this study, differential gene expression analysis was performed to identify expression differences between the control and RG6016-treated samples. As a result of the analysis, a total of 961 genes were found to be significantly differentially expressed; among them, 541 genes were upregulated and 526 genes were downregulated (Figure 6A). As shown in Figure 6B, the differentially expressed genes (DEGs) are distributed according to their log_2_ fold change and adjusted *p*-values. The upregulated genes exhibited statistically significant positive fold changes, while the downregulated genes showed statistically significant negative fold changes. In addition, 17,472 genes were identified as not significantly differentially expressed. Some genes showed log_2_ fold change values greater than 6, indicating marked differences in gene expression levels between the control and RG6016-treated groups. The findings in Figure 6B demonstrate that the majority of both upregulated and downregulated genes exhibit not only statistically significant *p*-values but also substantial fold changes. These data strongly support that RG6016 treatment has a significant and notable impact on gene expression.

### 3.3. DEG2 Profiles Across All PDX Samples Following RG6016 Treatment

**Figure 7:**
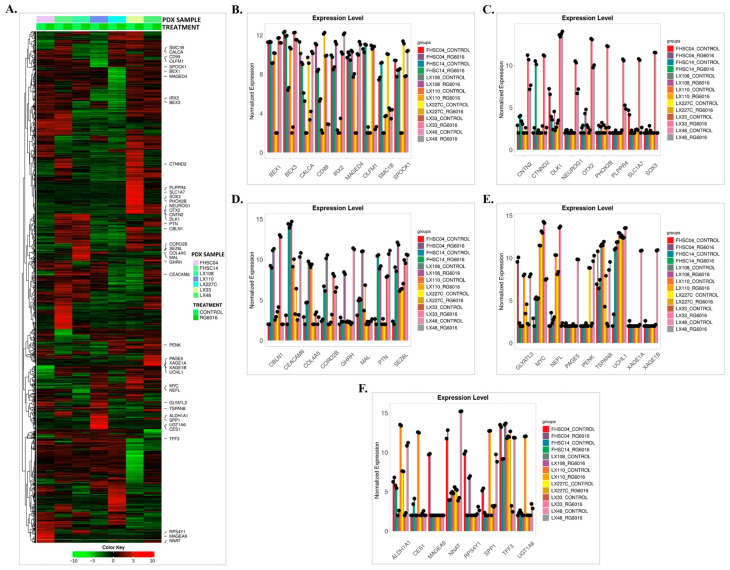
Heatmap and expression profiles of key genes. (**A**) The heatmap displays gene expression levels in PDX samples treated with control or RG6016. The colored bars at the top represent different PDX samples and treatment groups. Green indicates downregulation, while red indicates upregulation. Genes are ordered using hierarchical clustering based on their expression levels across conditions. (**B**–**F**) The bar graphs show the normalized expression levels of selected genes. Black dots represent biological replicates, and error bars indicate standard deviation.

The heatmap presented in Figure 7A reveals statistically significant differences in gene expression profiles across various PDX samples (FHSC04, FHSC14, LX110, LX227C, LX33, LX48, and LX108) and treatment conditions (control and RG6016). Hierarchical clustering analysis was applied to identify gene expression patterns and demonstrated that RG6016 treatment led to distinct expression profiles in the treated PDX samples. In addition, the associated bar graphs (Figure 7B–F) visualize the normalized gene expression levels in the control and RG6016-treated PDX samples. Overall, RG6016 treatment caused notable changes in gene expression levels across all samples, with PDX model-specific variations in response. According to the differential expression analysis, RG6016 induced statistically significant changes in gene expression in certain PDX samples. As shown in Figure 7B, *CALCA* (*p* = 0.048), *CD99* (*p* = 0.007), *IRX2* (*p* < 0.001), *SPOCK1* (*p* = 0.006), and *OLFM1* (*p* = 0.009) were significantly downregulated in LX110. In LX227C, *BEX1* (*p* = 0.01), *BEX3* (*p* = 0.031), and *MAGED4* (*p* = 0.027) were also significantly downregulated under RG6016 treatment, while *SMC1B* (*p* = 0.023) was significantly upregulated. Figure 7C shows that in LX33, *CNTN2* (*p* = 0.021), *CTNND2* (*p* = 0.029), *NEUROG1* (*p* = 0.005), *OTX2* (*p* = 0.03), *PHOX2B* (*p* < 0.001), *PLPPR4* (*p* = 0.002), *SLC1A7* (*p* = 0.031), and *SOX3* (*p* = 0.004) were significantly upregulated. Additionally, *DLK1* (*p* = 0.003) showed significantly increased expression in LX48 following RG6016 treatment. As shown in Figure 7D, *CBLN1* (*p* = 0.004) was upregulated in LX33; *CEACAM6* (*p* < 0.001), *COL4A5* (*p* = 0.01), *CORO2B* (*p* = 0.03), and *MAL* (*p* = 0.007) were upregulated in LX108; *GHRH* (*p* = 0.01) and *PTN* (*p* = 0.045) in LX48; and *SEZ6L* (*p* = 0.04) in FHSC04. Conversely, *MYC* (*p* < 0.001) and *UCHL1* (*p* = 0.02) were significantly downregulated in FHSC04, and *TSPAN8* (*p* = 0.01) in LX33 under RG6016 treatment. Additionally, Figure 7E shows that *GLYATL3* (*p* = 0.035) was significantly upregulated in FHSC04, *NEFL* (*p* = 0.009) in LX33, and *PAGE5* (*p* = 0.003), *XAGE1A* (*p* = 0.014), and *XAGE1B* (*p* = 0.03) in LX48. In Figure 7F, *ALDH1A1* (*p* = 0.015), *CES1* (*p* = 0.03), *SPP1* (*p* = 0.007), and *UGT1A6* (*p* = 0.003) were significantly upregulated in LX110, while *MAGEA9* (*p* = 0.018) and *RPS4Y1* (*p* = 0.008) were upregulated in FHSC04, and *NNAT* (*p* < 0.001) in LX48. In contrast, *TFF3* (*p* < 0.001) was significantly downregulated in LX33.

These findings demonstrate that RG6016 treatment induces distinct, statistically significant changes in gene expression in a PDX sample-specific manner. The data highlight that the effects of RG6016 are not limited to a general response but involve strong and specific molecular reactions in certain PDX samples (see Table 3).

**Figure 8:**
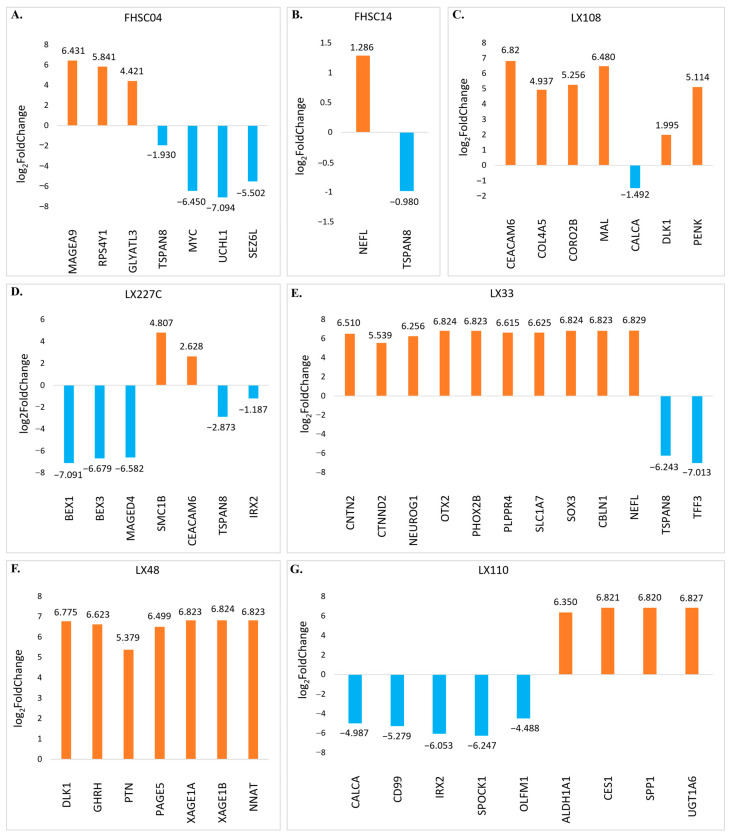
PDX sample-specific differential gene expression (Log_2_FoldChange). Bar graphs display the log_2_ fold change values of DEGs in various PDX samples following RG6016 treatment. Positive log_2_ fold change values (orange bars) represent genes with significantly increased expression, while negative log_2_ fold change values (blue bars) indicate genes with significantly decreased expression. Expression changes in response to RG6016 treatment were determined for the following PDX samples: (**A**) FHSC04, (**B**) FHSC14, (**C**) LX108, (**D**) LX227C, (**E**) LX33, (**F**) LX48, and (**G**) LX110.

Figure 8 presents the results of differential gene expression analysis across different PDX samples following RG6016 treatment. In each graph (A–G), genes that were significantly upregulated (orange bars) and downregulated (blue bars) based on their log_2_FoldChange values are displayed. In FHSC04 (Figure 8A), the most prominently upregulated genes were *MAGEA9*, *RPS4Y1*, and *GLYATL3*, while *MYC*, *UCHL1*, and *SEZ6L* were among the most strongly downregulated. In FHSC14 (Figure 8B), *NEFL* showed a mild upregulation, whereas *TSPAN8* was downregulated. The LX108 sample (Figure 8C) exhibited strong upregulation of genes such as *CEACAM6*, *COL4A5*, and *CORO2B*, while *TSPAN8* was downregulated. In the LX227C (Figure 8D), *BEX1*, *BEX3*, and *MAGED4* were the most prominently downregulated genes, whereas *CEACAM6* and *TSPAN8* were upregulated. In the LX33 (Figure 8E), genes such as *CNTN2*, *NEUROG1*, *OTX2*, and *PHOX2B* were notably upregulated, while *TSPAN8* and *TFF3* showed strong downregulation. In LX48 (Figure 8F), *DLK1*, *GHRH*, and *PTN* were upregulated. Lastly, in LX110 (Figure 8G), *CES1*, *SPP1*, and *UGT1A6* were among the most strongly upregulated genes, while *CALCA* (log_2_FoldChange: −4.987), *CD99*, and *IRX2* were significantly downregulated. The differential expression analyses presented in Figure 8 clearly demonstrate that RG6016 treatment induces statistically significant changes in gene expression levels across all PDX samples. When evaluated in terms of both upregulation and downregulation, several genes showed notably strong responses to the treatment. These genes exhibited remarkably high log_2_FoldChange values and levels of statistical significance compared to others. This highlights that the biological effects of RG6016 are not only widespread but also specific and potent, emphasizing the depth of PDX tumor-specific molecular responses and their potential clinical relevance.

### 3.4. Pathway Enrichment Analysis of DEGs

To understand the effects of RG6016 treatment on biological processes and signaling pathways across different PDX samples, pathway enrichment analysis was performed using the DEGs. Table 4 and Figure S1 illustrate the biological pathways associated with both upregulated and downregulated genes, along with their statistical significance levels.

#### 3.4.1. Upregulated Pathways

Following RG6016 treatment, the most prominently activated biological pathways were identified as nicotine addiction (Fold Enrichment = 4.09, −log_10_(FDR) = 4.23, *n* = 16), and protein digestion and absorption (Fold Enrichment = 3.26, −log_10_(FDR) = 7.86, *n* = 35) (Table 4; Figure S1A,B). In addition, the extracellular matrix (ECM)–receptor interaction pathway (Fold Enrichment = 3.14, −log_10_(FDR) = 6.29, *n* = 30) was also identified among the upregulated biological processes following RG6016 treatment. Figure S1A presents the network analysis of the upregulated pathways, while Figure S1B ranks them according to their statistical significance. These findings reveal that RG6016 treatment activates specific biological pathways in a PDX sample-dependent manner. Notably, the upregulation of pathways such as ECM–receptor interaction, which are associated with cytoskeletal dynamics and extracellular matrix remodeling, points to the potential impact of RG6016 on cell architecture and interaction with the microenvironment. Furthermore, changes in pathways related to nicotine addiction, metabolism, and cellular signaling suggest that RG6016 may influence a broad spectrum of biological processes within treated PDX samples.

#### 3.4.2. Downregulated Pathways

The biological pathways suppressed following RG6016 treatment are presented in Table 4 and Figure S1C,D. The most prominently downregulated processes include histidine metabolism (Fold Enrichment = 3.52, −log_10_(FDR) = 2.87, *n* = 12) and steroid hormone biosynthesis (Fold Enrichment = 2.36, −log_10_(FDR) = 2.31, *n* = 19). In addition, xenobiotic metabolism by cytochrome P450 (Fold Enrichment = 2.29, −log_10_(FDR) = 2.87, *n* = 25) pathways were also found to be downregulated following RG6016 treatment. Figure S1C visualizes the interactions between these pathways in a network diagram, while Figure S1D presents a bar chart ranking the most significantly downregulated pathways based on their −log_10_(FDR) values. These results indicate that RG6016 treatment suppresses key metabolic processes and cellular components. In particular, notable decreases were observed in biological pathways such as histidine metabolism, steroid hormone biosynthesis, and ECM–receptor interaction, suggesting that RG6016 may exert its therapeutic effects, in part, by downregulating pathways critical to tumor cell metabolism, signaling, and extracellular communication.

It is noteworthy that certain biological pathways identified as enriched in this study, including taste transduction and nicotine addiction, might not represent a direct relevance to the biology of SCLC or to the genes TSPAN8, UCHL1, and MYC, which were identified as potential targets of RG6016 through our bioinformatic and molecular docking analyses. This may be attributed to the broad nature of the differentially expressed gene lists and the statistical characteristics of pathway enrichment analysis, which can occasionally highlight pathways of limited biological relevance due to gene overlaps or database annotations. Therefore, although the presence of such pathways is recognized, the interpretation of the findings is primarily centered on enrichment results that demonstrate biological relevance and are substantiated by the existing literature within the context of cancer biology and therapeutic targeting.

### 3.5. Network Analysis

**Figure 9:**
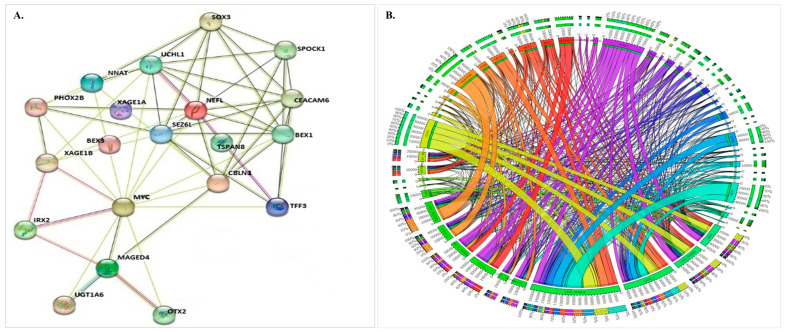
The PPI networks and Circos plot of DEGs in RG6016-treated PDX models. (**A**) The PPI network displays the top 20 statistically significant DEGs following RG6016 treatment and their biological interactions. Genes located at the center of the network exhibit strong interactions with other genes. (**B**) The Circos plot visualizes the complex interactions among the top 40 statistically significant DEGs. LSD1 was not included in the PPI network, as it did not appear in the DEG list. It was used solely as a reference target in the docking analysis.

Protein–protein interaction (PPI) network analysis was performed to investigate the relationships among DEGs across all PDX samples following RG6016 treatment, enabling the identification of interactions among significantly altered proteins and their potential impact on biological processes (Figure 9). The analysis revealed interactions among proteins encoded by genes such as *MYC*, *UCHL1*, and *TSPAN8*, forming a tightly connected network characterized by functionally interdependent and strongly interacting components (Figure 9A). In Figure 9B, a circular connection diagram visualizes the links between DEGs responsive to RG6016 treatment and their associated biological processes. This diagram shows that genes affected by the treatment are associated with a wide array of biological functions. The colored chords in the diagram indicate which functional categories each gene is linked to, with some genes being connected to multiple biological pathways. The fact that certain genes are involved in several functional categories implies that they may play a central role in modulating the cellular response to treatment. These findings support the widespread influence of RG6016 on intracellular signaling pathways and highlight the complexity of the molecular response induced by treatment.

### 3.6. Molecular Docking Analysis

In this study, molecular docking analyses were conducted to evaluate the potential interactions between the LSD1 inhibitor RG6016 and its target proteins in SCLC samples. In addition to the positive control LSD1, 13 proteins encoded by genes that were significantly downregulated as a result of LSD1 inhibition were selected as targets, based on differential expression analysis of RNA-seq data. The goal was to identify proteins that could be directly or indirectly affected by RG6016. Docking analyses were performed for a total of 14 proteins, including the known target LSD1/FAD complex. For each interaction, docking score (kcal/mol), conventional hydrogen bonds, interacting amino acid residues, and bond lengths were assessed. The general docking data are presented in Table S2, whereas proteins with binding energies equal to or lower than −7.0 kcal/mol, which indicate strong and potentially biologically relevant interactions, are detailed in Table 5. Proteins with weaker binding affinities (above −7.0 kcal/mol) are visualized in Figures S2–S4 and were excluded from prioritized interpretation.

**Figure 10:**
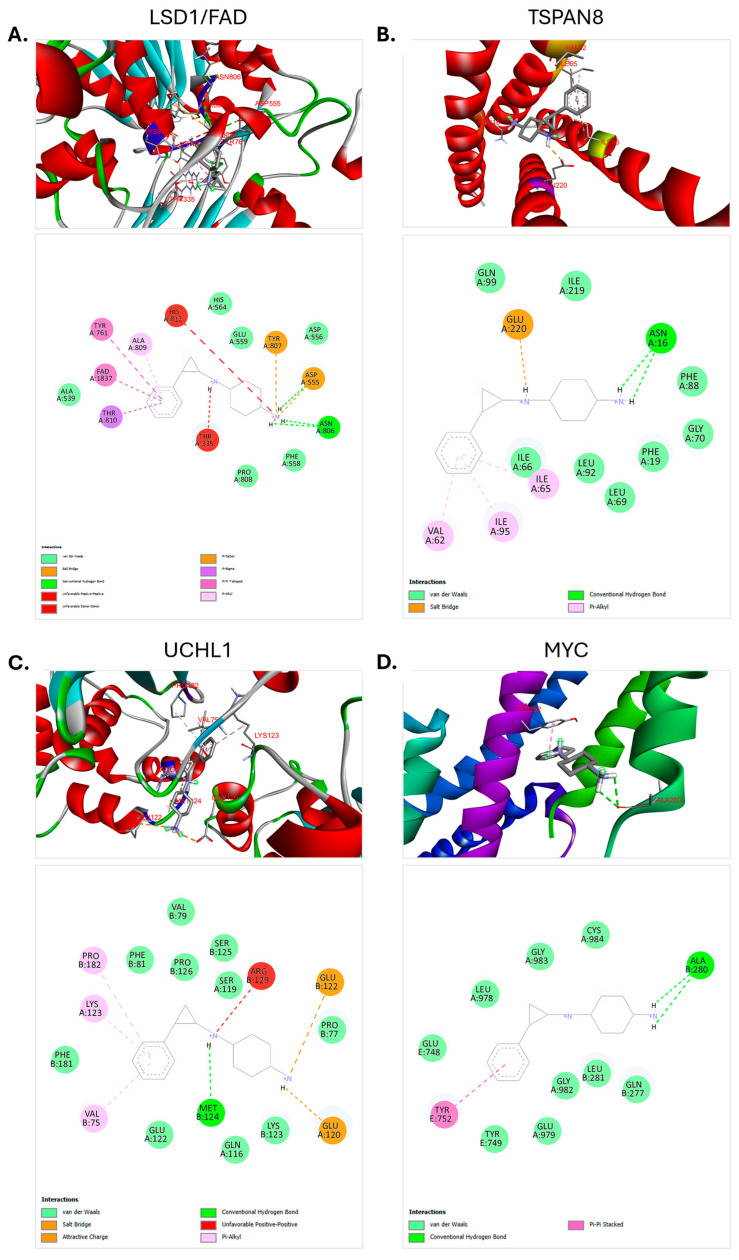
Binding poses and interaction diagrams of RG6016 with the LSD1/FAD complex (**A**), TSPAN8 (**B**), UCHL1 (**C**), and MYC (**D**) proteins. The upper panel displays the three-dimensional binding sites of RG6016 within each protein, while the lower panel presents two-dimensional interaction maps, including hydrogen bonds and hydrophobic interactions. In the LSD1 structure, the FAD cofactor is highlighted in purple to enhance visual distinction and biological interpretability. The FAD cofactor is clearly displayed in the upper panel of Figure 10A.

Among all proteins analyzed, LSD1, used as a positive control, exhibited a strong docking score with the ligand at −7.2 kcal/mol. This interaction involved three conventional hydrogen bonds with the amino acid residues ASP555 (2.21 Å) and ASN806 (2.57 and 2.60 Å). These findings confirm LSD1 as a direct and high-affinity target, in agreement with previously reported structural interactions. In addition to LSD1, three other proteins including TSPAN8 (−7.4 kcal/mol), UCHL1 (−7.2 kcal/mol), and MYC (−7.0 kcal/mol) also exhibited docking scores with RG6016. TSPAN8 formed two conventional hydrogen bonds with ASN16, at distances of 2.44 Å and 2.48 Å. UCHL1 established a single hydrogen bond with MET124 (2.54 Å). MYC formed two hydrogen bonds with ALA280, at distances of 2.63 Å and 2.79 Å. These interactions suggest that RG6016 can stably bind within the active pockets of these proteins, potentially modulating their function. Figure 10 presents the 3D binding poses and 2D interaction diagrams of RG6016 with LSD1, TSPAN8, UCHL1, and MYC. These visuals demonstrate not only hydrogen bonding but also complementary interactions such as hydrophobic contacts (e.g., Pi–alkyl, van der Waals), π–π stacking, and in some cases, salt bridges. The presence of these interactions further supports a favorable docking pose of RG6016 to its targets. In conclusion, in addition to LSD1, significant interactions were identified between RG6016 and the downregulated gene products TSPAN8, UCHL1, and MYC. These findings suggest that the molecular effects of RG6016 may extend beyond LSD1 inhibition, potentially involving the modulation of additional cellular pathways through interactions with these target proteins.

On the other hand, other downregulated gene products such as OLFM1, MAGED4, SPOCK1, CALCA, TFF3, BEX1, BEX3, CD99, and IRX2 were not included in the core analysis of this study, as their docking scores ranged between −6.8 and −4.4 kcal/mol (Table S2). However, the docking analyses of these proteins revealed various binding motifs, including conventional hydrogen bonds, π–π stacking, cation–π interactions, and hydrophobic contacts, indicating that RG6016 may also potentially interact with these proteins (Figures S2–S4). These findings suggest that RG6016 may exhibit a multi-target binding profile in silico and that interactions observed with targets beyond LSD1 are also scientifically noteworthy. The potential interactions of RG6016 extending beyond LSD1 inhibition imply a broadened therapeutic profile for this compound. In conclusion, these in silico results, when supported by future experimental validations, may contribute to a more comprehensive understanding of the target engagement capacity of RG6016.

## 4. Discussion

SCLC accounts for approximately 15% of all lung cancers and is the leading cause of cancer-related deaths in men and the second most common cause in women worldwide [29,30]. Characterized by biological features such as rapid proliferation, high vascularity, and apoptotic imbalance, SCLC is also notable for its tendency to metastasize at an early stage [31,32]. In treatment-resistant tumors such as SCLC, LSD1 inhibition has been shown to trigger distinct cellular responses [11]. LSD1 specifically removes mono- and dimethyl groups from lysine 4 or lysine 9 on histone H3 (H3K4me1/2 and H3K9me1/2), thereby acting as either a transcriptional repressor or activator depending on the context [33]. Another critical function of LSD1 is its role in hypoxia response [34]. Under hypoxic conditions, stabilization and activation of HIF-1α lead to the transcriptional induction of various target genes [34,35]. Pharmacological inhibition of LSD1 has been shown to delay tumor growth and exert cytostatic effects in both in vitro and xenograft models [11]. LSD1 inhibitor therapy has made progress in the treatment of hematological cancers [36] and lung cancer [11]. Studies on the role of LSD1 in prostate cancer have demonstrated that LSD1 inhibitors suppress MYC signaling and reduce tumor growth [37]. RG6016 is a potent LSD1 inhibitor that exerts its effect by irreversibly binding to the FAD cofactor of LSD1. It is currently undergoing clinical trials for the treatment of AML and solid tumors [18].

In this study, our bioinformatics analyses demonstrated that RG6016 induces selective, PDX tumor model-specific, and statistically significant changes in gene expression. The downregulation of genes such as *MYC*, *UCHL1*, and *TSPAN8*, along with the high binding affinities observed between RG6016 and the proteins encoded by these genes, suggests that these targets may play key roles in the therapeutic response. As LSD1 primarily functions as a transcriptional repressor, our focus was directed toward downregulated genes to better reflect the direct consequences of its inhibition. Pathway enrichment analyses revealed that RG6016 affects pathways related to cytoskeletal organization, ECM interactions, and cellular metabolism. Notably, the activation of pathways such as ECM–receptor interaction, and the suppression of pathways like histidine metabolism and steroid hormone biosynthesis, point to the multifaceted biological effects of the drug. However, some statistically enriched pathways identified in the analysis may not be directly relevant to the biological context of the study, likely due to gene overlap and the inherent limitations of enrichment algorithms. PPI analyses further indicated that the gene products targeted by RG6016 are involved in numerous biological processes, supporting the notion that its effects are mediated through a broad molecular network. In molecular docking analyses, LSD1 was evaluated as a positive control and exhibited favorable docking pose with RG6016. Similarly, high binding affinities were observed with TSPAN8, UCHL1, and MYC proteins. The formation of hydrogen bonds and complementary interactions with these proteins suggests that RG6016 may potentially modulate their functions, highlighting its broader therapeutic impact beyond LSD1 inhibition.

TSPAN8 is a transmembrane protein that organizes tetraspanin-enriched microdomains on the cell membrane, thereby contributing to the spatial arrangement of membrane proteins [38]. Through this function, it plays a critical role in the regulation of various fundamental cellular processes, including cell–cell interactions and signal transduction. The influence of TSPAN8 on cell motility is particularly important in the context of cancer progression. Elevated *TSPAN8* mRNA expression has been reported in several cancer types, including colorectal, pancreatic [39], gastric [40], and hepatocellular carcinomas [41], as well as in melanoma [42] and glioma [43]. UCHL1 is a multifunctional protein and a key member of the deubiquitination protein family. It regulates cellular proliferation, differentiation, and damage response by modulating both ubiquitin-dependent and -independent pathways [44,45]. Studies have shown that UCHL1 is upregulated in lung cancers and plays a critical regulatory role in tumorigenesis [46,47]. Other cancers with high UCHL1 expression include breast cancer [48,49,50], melanoma [51,52], and osteosarcoma [53,54]. MYC is a potent transcription factor that regulates essential processes such as cell growth, cell cycle progression, metabolism, and apoptosis, making it a central player in cancer biology [55,56,57,58,59]. The overexpression of *MYC* is a hallmark of many cancer types and has been shown to directly initiate malignant transformation in various malignancies [56]. In SCLC, the overexpression of MYC family genes contributes to disease progression by influencing cell cycle and apoptosis pathways [60].

Interestingly, LSD1 has also been shown to enhance the stability of the transcription factor HIF-1α by demethylating both HIF-1α and RACK1 [61,62,63,64]. Similarly, UCHL1 contributes to the stabilization of HIF-1α by deubiquitinating it, thereby supporting cellular adaptation to hypoxia [65]. MYC, in collaboration with hypoxia-related HIF-1α, has been shown to promote glycolysis and suppress mitochondrial respiration, effectively reprogramming cellular metabolism. Moreover, MYC stabilizes HIF-1α under normoxic conditions and enhances its accumulation under hypoxic conditions, establishing a strong synergy between these two factors in support of metabolic reprogramming [66]. Additionally, LSD1 inhibitors have been reported to suppress MYC signaling and reduce tumor growth [67]. In line with these findings, our results showed that the expression levels of *MYC* and *UCHL1* were significantly downregulated following RG6016 treatment. This suggests that RG6016 may exert its effects on cellular proliferation and metabolism through these targets. Based on these data, it is proposed that RG6016 may modulate the HIF-1α pathway via LSD1, UCHL1, and MYC in aggressive cancers such as SCLC, thereby influencing key processes like hypoxia response, metastasis, and neuroendocrine differentiation. With these multifaceted effects, RG6016 appears to be a promising therapeutic candidate capable of targeting critical pathways in tumor biology.

This study evaluated the selective efficacy of the LSD1 inhibitor RG6016 in SCLC and the molecular mechanisms underlying this effect through bioinformatics analyses and molecular docking approaches. The bioinformatic analysis of RNA-seq data revealed altered gene expression profiles in response to RG6016 treatment, while docking results demonstrated favorable docking pose between RG6016 and the gene products of *TSPAN8*, *UCHL1*, and *MYC*. These findings suggest that the effects of RG6016 may extend beyond LSD1 inhibition, potentially influencing multiple cellular networks and pathways. Given the critical roles of TSPAN8, UCHL1, and MYC in key biological processes in aggressive cancers like SCLC, targeting these molecules may offer valuable therapeutic potential. In this study, in silico molecular docking analysis revealed strong predicted interactions between RG6016 and several proteins not previously associated with FAD-dependent activity, including UCHL1, MYC, and TSPAN8. Although the docking scores with TSPAN8, UCHL1, and MYC were relatively high, these findings do not imply definitive binding or reactivity. Rather, they suggest that these proteins may warrant further experimental investigation to explore their potential modulation by RG6016. While these findings suggest a possible multi-target profile for RG6016, it is important to acknowledge that computational docking inherently lacks the capacity to fully capture the dynamic conformational flexibility of proteins under physiological conditions. Additionally, factors such as membrane localization (particularly relevant for tetraspanins like TSPAN8) and post-translational modifications are typically not incorporated into standard docking models. Therefore, further experimental validation is required to determine whether these predicted interactions are biologically relevant. While this study did not include molecular dynamics (MD) simulations due to its exploratory in silico design, future work will aim to evaluate the stability of the identified RG6016–protein complexes through MD-based MM/PBSA analysis. This study is limited by its in silico approach. While molecular docking provides valuable predictions regarding ligand–protein interactions, it does not account for conformational flexibility, post-translational modifications, membrane localization, or the thermodynamic profile of binding under physiological conditions. Furthermore, the absence of MD simulations and MM/PBSA binding energy calculations restricts the precision of interaction assessments. Experimental validation of the identified targets will be essential to confirm their biological relevance. Further studies using cellular models or proteomics approaches will be essential to clarify the extent effects of RG6016, which may provide new insights into its mechanism of action or reveal previously unrecognized therapeutic opportunities. In future analyses, the inclusion of upregulated genes is also planned, as their investigation may reveal clinically relevant pathways of potential activation. Further investigation into the molecular mechanisms and therapeutic impact of RG6016 in SCLC is of great importance for future research and clinical translation.

## 5. Conclusions

In conclusion, this study comprehensively revealed the selective efficacy of the LSD1 inhibitor RG6016 in SCLC and its potential molecular targets through bioinformatic and molecular docking analyses. The downregulation of genes such as *MYC*, *UCHL1*, and *TSPAN8*, along with their potential in silico interaction with RG6016, suggests that the compound may possess a multifaceted mechanism of action. Furthermore, the potential involvement of these targets in key processes such as hypoxia, metabolism, and tumor progression through HIF-1α signaling further supports the therapeutic potential of RG6016 in tumor biology. However, to confirm the reliability and biological relevance of these findings, further in vitro and in vivo experimental validation is essential.

## Figures and Tables

**Table 1:** Experimental design.

Sample Name	PDX Sample	Treatment
FHSC04_Control	FHSC04	CONTROL
FHSC04_RG6016	FHSC04	RG6016
FHSC14_Control	FHSC14	CONTROL
FHSC14_RG6016	FHSC14	RG6016
LX108_Control	LX108	CONTROL
LX108_RG6016	LX108	RG6016
LX110_Control	LX110	CONTROL
LX110_RG6016	LX110	RG6016
LX227C_Control	LX227C	CONTROL
LX227C_RG6016	LX227C	RG6016
LX33_Control	LX33	CONTROL
LX33_RG6016	LX33	RG6016
LX48_Control	LX48	CONTROL
LX48_RG6016	LX48	RG6016

**Table 2:** Center coordinates and grid box dimensions of proteins used in docking analyses.

Protein	Center at (X, Y, Z)	Dimension (Å)
LSD1/FAD complex	X: −5.864, Y: 60.314, Z: 93.813	40 Å × 40 Å × 40 Å
TSPAN8	X: −35.139, Y: −19.879, Z: 50.937	100 Å × 100 Å × 100 Å
UCHL1	X: 84.171, Y: 25.697, Z: 19.341	40 Å × 40 Å × 40 Å
MYC	X: 53.448, Y: 47.445, Z: 58.079	40 Å × 40 Å × 40 Å
BEX1	X: −35.261, Y: −20.238, Z: 50.826	100 Å × 100 Å × 100 Å
BEX3	X: 74.024, Y: 0.969, Z: −16.177	100 Å × 100 Å × 100 Å
CALCA	X: −5.523, Y: −2.272, Z: 0.537	100 Å × 100 Å × 100 Å
CD99	X: −1.135, Y: −2.164, Z: −6.97	100 Å × 100 Å × 100 Å
IRX2	X: −4.002, Y: 0.413, Z: −4.138	40 Å × 40 Å × 40 Å
MAGED4	X: −2.805, Y: 5.328, Z: −3.031	40 Å × 40 Å × 40 Å
OLFM1	X: 4.006, Y: 1.697, Z: −2.998	40 Å × 40 Å × 40 Å
SEZL6	X: −6.505, Y: 3.144, Z: 16.417	100 Å × 100 Å × 100 Å
TFF3	X: −1.078, Y: 2.169, Z: −4.762	40 Å × 40 Å × 40 Å
SPOCK1	X: 0.809, Y: 3.861, Z: −0.615	40 Å × 40 Å × 40 Å

**Table 3:** Gene expression patterns following RG6016 treatment based on DEG2 results.

Up/Downregulated	Ensembl ID	Symbol	Entrez-Gene ID	log_2_FC Values	Description
Up	ENSG00000277586	NEFL	4747	6.829	Neurofilament light chain
Up	ENSG00000167165	UGT1A6	54578	6.827	UDP glucuronosyltransferase family 1 member A6
Up	ENSG00000086548	CEACAM6	4680	6.825	CEA cell adhesion molecule 6
Up	ENSG00000134595	SOX3	6658	6.824	SRY-box transcription factor 3
Up	ENSG00000204382	XAGE1B	653067	6.824	X antigen family member 1B
Up	ENSG00000165588	OTX2	5015	6.824	Orthodenticle homeobox 2
Up	ENSG00000102924	CBLN1	869	6.823	Cerebellin 1 precursor
Up	ENSG00000204379	XAGE1A	653220	6.823	X antigen family member 1A
Up	ENSG00000109132	PHOX2B	8929	6.823	Paired like homeobox 2B
Up	ENSG00000053438	NNAT	4826	6.823	Neuronatin
Up	ENSG00000198848	CES1	1066	6.821	Carboxylesterase 1
Up	ENSG00000118785	SPP1	6696	6.820	Secreted phosphoprotein 1
Up	ENSG00000185559	DLK1	8788	6.775	Delta like non-canonical Notch ligand 1
Up	ENSG00000162383	SLC1A7	6512	6.625	Solute carrier family 1 member 7
Up	ENSG00000118702	GHRH	2691	6.623	Growth hormone releasing hormone
Up	ENSG00000117600	PLPPR4	9890	6.615	Phospholipid phosphatase related 4
Up	ENSG00000184144	CNTN2	6900	6.510	Contactin 2
Up	ENSG00000158639	PAGE5	90737	6.499	PAGE family member 5
Up	ENSG00000172005	MAL	4118	6.480	Mal, T cell differentiation protein
Up	ENSG00000123584	MAGEA9	4108	6.431	MAGE family member A9
Up	ENSG00000165092	ALDH1A1	216	6.350	Aldehyde dehydrogenase 1 family member A1
Up	ENSG00000181965	NEUROG1	4762	6.256	Neurogenin 1
Up	ENSG00000129824	RPS4Y1	6192	5.841	Ribosomal protein S4 Y-linked 1
Up	ENSG00000169862	CTNND2	1501	5.539	Catenin delta 2
Up	ENSG00000105894	PTN	5764	5.379	Pleiotrophin
Up	ENSG00000103647	CORO2B	10391	5.256	Coronin 2B
Up	ENSG00000181195	PENK	5179	5.114	proenkephalin
Up	ENSG00000188153	COL4A5	1287	4.937	Collagen type IV alpha 5 chain
Up	ENSG00000077935	SMC1B	27127	4.807	Structural maintenance of chromosomes 1B
Up	ENSG00000203972	GLYATL3	389396	4.421	Glycine-N-acyltransferase like 3
Down	ENSG00000130558	OLFM1	10439	−4.488	Olfactomedin 1
Down	ENSG00000110680	CALCA	796	−4.987	Calcitonin related polypeptide alpha
Down	ENSG00000002586	CD99	4267	−5.279	CD99 molecule (Xg blood group)
Down	ENSG00000100095	SEZ6L	23544	−5.502	Seizure related 6 homolog like
Down	ENSG00000170561	IRX2	153572	−6.053	Iroquois homeobox 2
Down	ENSG00000127324	TSPAN8	7103	−6.243	Tetraspanin 8
Down	ENSG00000152377	SPOCK1	6695	−6.247	SPARC (osteonectin), cwcv and kazal like domains proteoglycan 1
Down	ENSG00000136997	MYC	4609	−6.450	MYC proto-oncogene, bHLH transcription factor
Down	ENSG00000154545	MAGED4	728239	−6.582	MAGE family member D4
Down	ENSG00000166681	BEX3	27018	−6.679	Brain expressed X-linked 3
Down	ENSG00000160180	TFF3	7033	−7.013	Trefoil factor 3
Down	ENSG00000133169	BEX1	55859	−7.091	Brain expressed X-linked 1
Down	ENSG00000154277	UCHL1	7345	−7.094	Ubiquitin C-terminal hydrolase L1

**Table 4:** Pathway enrichment analysis results for RG6016-treated PDX samples.

Direction	DEG2 Analysis: All PDX Sample Pathways	FoldEnriched	nGenes	−log_10_(FDR)
Up	Nicotine addiction	4.09	16	4.23
	Protein digestion and absorption	3.26	35	7.86
	ECM-receptor interaction	3.14	30	6.29
Down	Histidine metabolism	3.52	12	2.87
	Steroid hormone biosynthesis	2.36	19	2.31
	Metabolism of xenobiotics by cytochrome P450	2.29	25	2.87

**Table 5:** Molecular docking results of RG6016 with positive control LSD1 and proteins showing docking scores.

Protein	Docking Score (Kcal/mol)	Amino Acid Interaction	Hydrogen BondDistance (Å)	Number of Conventional Hydrogen Bonds
LSD1/FAD complex	−7.2	ASP555	2.21	3
		ASN806	2.57	
		ASN806	2.60	
TSPAN8	−7.4	ASN16	2.44	2
		ASN16	2.48	
UCHL1	−7.2	MET124	2.54	1
MYC	−7.0	ALA280	2.63	2
		ALA280	2.79	

## Data Availability

The data are available from the authors on reasonable request.

## Supplementary Materials

The following supporting information can be downloaded at: https://www.mdpi.com/article/10.3390/bioengineering12050504/s1, Pathway enrichment analysis of DEGs in RG6016-treated PDX samples. (**A**) Network graph of downregulated pathways visualizing genetic connections among pathways (Figure S1). General view of docking position of RG6016 in binding sites of MAGED4, OLFM1, TFF3, and SPOCK1 is presented (Figure S2), followed by molecular docking analyses of RG6016 with CALCA, CD99, IRX2, and SEZ6L (Figure S3), and with BEX1 and BEX3 (Figure S4). A summary of AlphaFold-derived models, including UniProt IDs and average pLDDT scores, is provided, noting that some lower-confidence models were included due to lack of alternative human structures (Table S1). Comprehensive docking data, including binding energies and interaction details for all 14 analyzed proteins, are provided (Table S2).
