# Supplementary material for: In Silico Identification of LSD1 Inhibition-Responsive Targets in Small Cell Lung Cancer
Source: Bioengineering (Basel). 2025 May 10;12(5):504. doi: 10.3390/bioengineering12050504 (PMC12108737; doi:10.3390/bioengineering12050504)
Supplement: Supplementary file 1 [file bioengineering-12-00504-s001.zip › bioengineering-3586110-Supplementary materials.pdf]

**Table S.1.** AlphaFold IDs, UniProt accessions, and average pLDDT (Predicted Local Distance Difference Test) scores of target proteins analyzed in relation to LSD1 inhibitor RG6016.

| AlphaFold ID | Protein | UniProt | Average pLDDT |
|---|---|---|---|
| AF-P19075-F1-v4 | TSPAN8 | P19075 | 88.12 (High) |
| AF-Q9HBH7-F1-v4 | BEX1 | Q9HBH7 | 66.95 (Low) |
| AF-Q00994-F1-v4 | BEX3 | Q00994 | 64.32 (Low) |
| AF-P01258-F1-v4 | CALCA | P01258 | 64.56 (Low) |
| AF-P14209-F1-v4 | CD99 | P14209 | 60.72 (Low) |
| AF-Q9BZI1-F1-v4 | IRX2 | Q9BZI1 | 54.11 (Low) |
| AF-Q96JG8-F1-v4 | MAGED4 | Q96JG8 | 53.72 (Low) |
| AF-Q99784-F1-v4 | OLFM1 | Q99784 | 79.39 (High) |
| AF-Q9BYH1-F1-v4 | SEZ6L | Q9BYH1 | 68.48 (Low) |
| AF-Q07654-F1-v4 | TFF3 | Q07654 | 80.13 (High) |
| AF-Q08629-F1-v4 | SPOCK1 | Q08629 | 64.56 (Low) |

**Table S.2.** Molecular docking data of RG6016 (ORY-1001) with 14 analyzed proteins. This table lists the docking results for all 14 proteins analyzed with RG6016, including binding energies, hydrogen bond interactions, and additional interaction details where applicable.

| Protein | Docking Score (Kcal/mol) | Aminoacid Interaction | Hydrogen Bond Distance (Å) | Number of conventional hydrogen bonds |
|---|---|---|---|---|
| LSD1/FAD complex | -7.2 | ASP555 | 2,21 | 3 |
| | | ASN806 | 2,57 | |
| | | ASN806 | 2,60 | |
| | | | | |
| TSPAN8 | -7.4 | ASN16 | 2,44 | 2 |
| | | ASN16 | 2,48 | |
| | | | | |
| UCHL1 | -7.2 | MET124 | 2,54 | 1 |
| | | | | |
| MYC | -7.0 | ALA280 | 2,63 | 2 |
| | | ALA280 | 2,79 | |
| | | | | |
| BEX1 | -4.9 | MET67 | 2,28 | 1 |
| | | | | |
| BEX3 | -4.4 | TRP49 | 3,02 | 1 |
| | | | | |
| CALCA | -5.0 | PHE3 | 2,05 | 1 |
| | | | | |
| CD99 | -5.2 | – | – | – |

| | | | | |
|---|---|---|---|---|
| | | | | |
| IRX2 | -6.3 | – | – | – |
| | | | | |
| MAGED4 | -6.7 | LYS427 | 2,60 | 2 |
| | | PRO433 | 2,29 | |
| | | | | |
| OLFM1 | -6.6 | ASP360 | 2,86 | 5 |
| | | ASP360 | 2,95 | |
| | | GLU361 | 2,28 | |
| | | PRO456 | 2,44 | |
| | | PRO456 | 2,72 | |
| | | | | |
| SEZL6 | -6.0 | – | – | – |
| | | | | |
| TFF3 | -6.2 | TYR58 | 2,29 | 1 |
| | | | | |
| SPOCK1 | -6.2 | CYS304 | 2,20 | 2 |
| | | SER208 | 1,86 | |

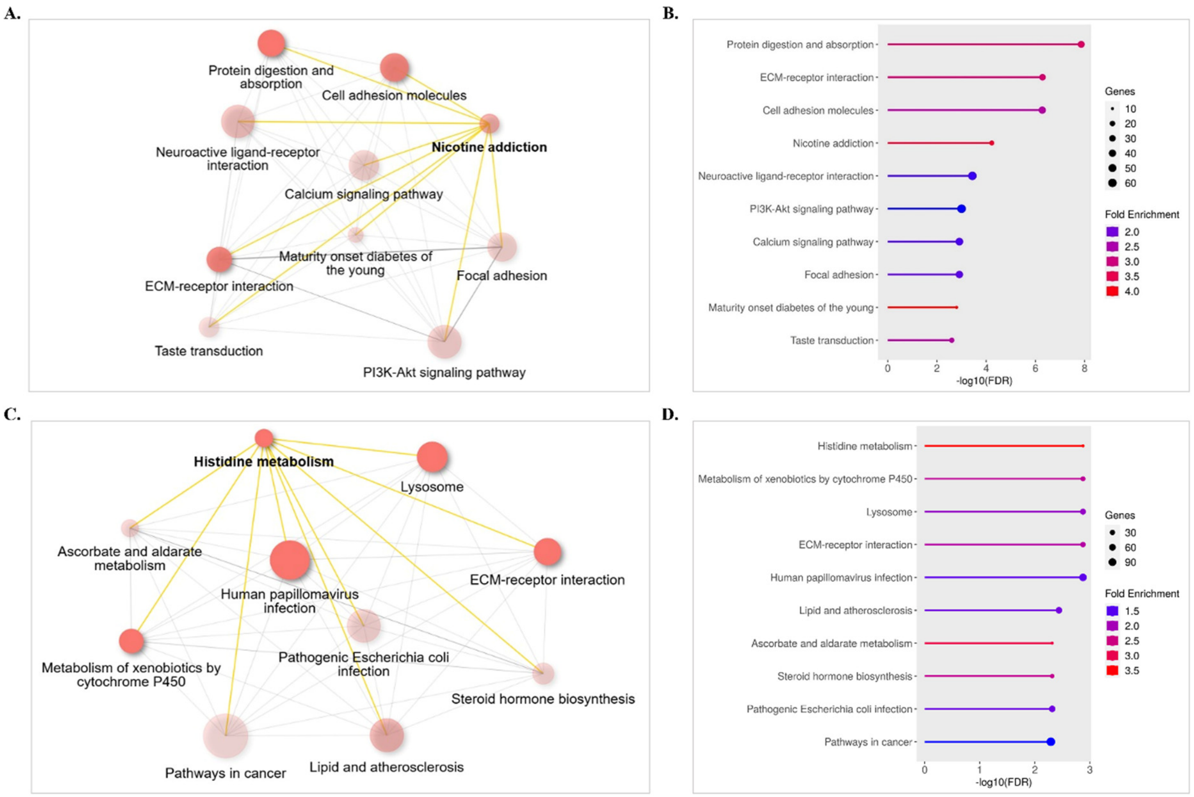


**Figure S.1.** Pathway enrichment analysis of DEGs in RG6016-treated PDX samples. **(A)** Network graph of downregulated pathways visualizing genetic connections among pathways. Node size represents relative importance of each pathway, while edges indicate shared genes between pathways. **(B)** Bar plot of top 10 downregulated pathways ranked by $-\log_{10}$(FDR) values. Colors represent fold enrichment levels. **(C)** Network graph of upregulated pathways illustrating relationships among biological processes activated following RG6016 treatment. **(D)** Bar plot of top 10 upregulated pathways ranked by $-\log_{10}$(FDR) values and color-coded based on fold enrichment.

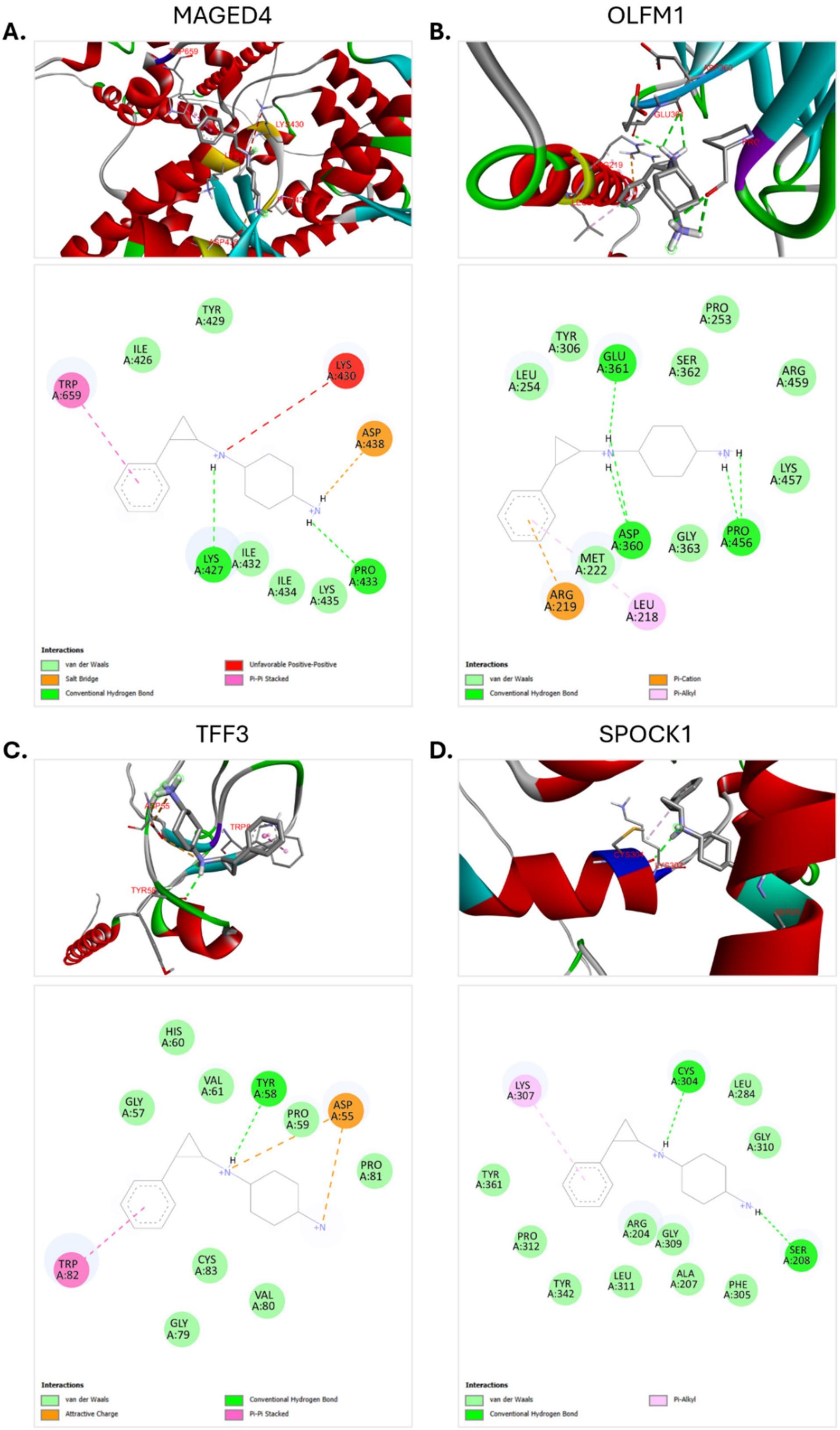


**Figure S.2.** A visualization of the molecular docking interactions between RG6016 (ORY-1001) and MAGED4 **(A)**, OLFM1 **(B)**, TFF3 **(C)**, and SPOCK1 **(D)** proteins. The upper panel shows the three-dimensional binding poses of RG6016 within each protein's active site, while the lower panel displays two-dimensional interaction diagrams, including hydrogen bonds, hydrophobic interactions, salt bridges, and other complementary binding interactions.

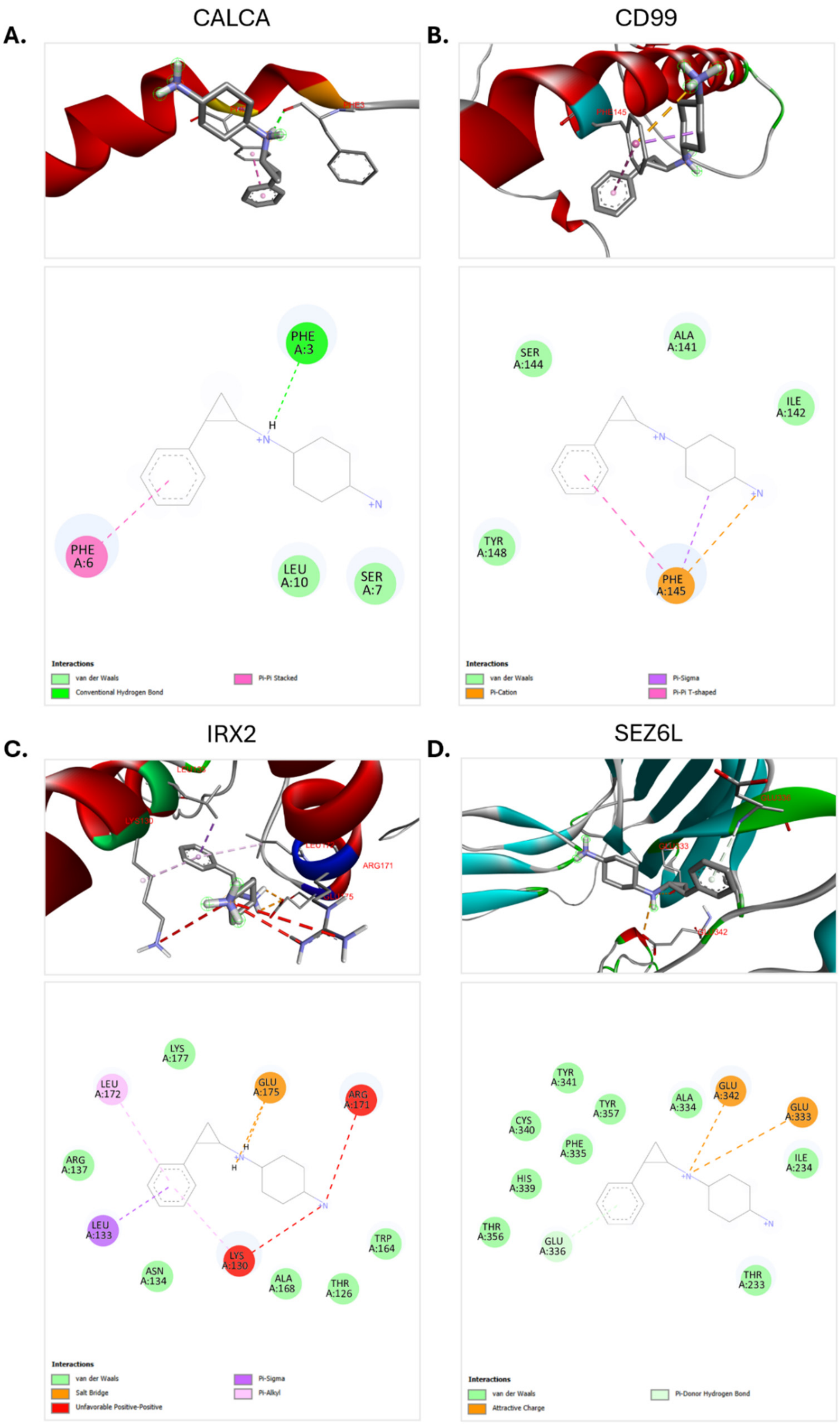


**Figure S.3.** A visualization of the molecular docking interactions between RG6016 (ORY-1001) and CALCA **(A)**, CD99 **(B)**, IRX2 **(C)**, and SEZ6L **(D)** proteins. The upper panel displays the three-dimensional binding poses of RG6016 within the binding sites of each protein. The lower panel presents two-dimensional interaction diagrams, illustrating hydrogen bonds, hydrophobic interactions, π interactions, and other complementary binding types.

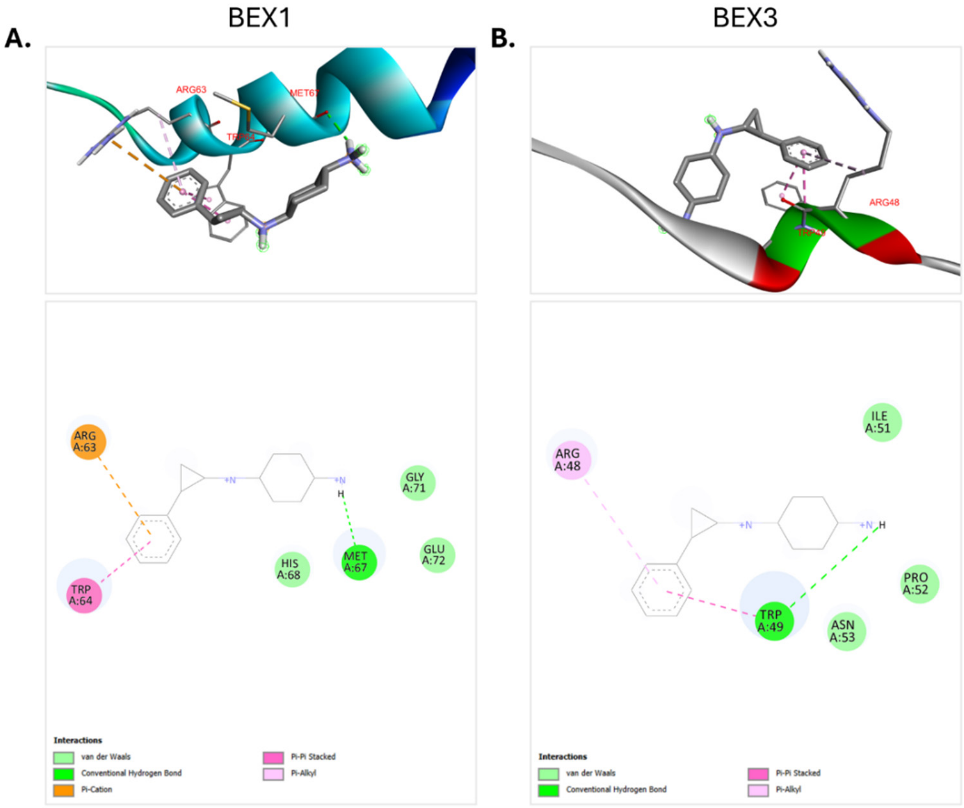


**Figure S.4.** A visualization of the molecular docking interactions between RG6016 (ORY-1001) and BEX1 **(A)** and BEX3 **(B)** proteins. The upper panel shows the three-dimensional binding positions, while the lower panel presents two-dimensional interaction maps including hydrogen bonds, π interactions, and van der Waals interactions.
